# Supplementary material for: Husbands’ knowledge and attitudes regarding postpartum depression
Source: PeerJ. 2025 May 13;13:e19426. doi: 10.7717/peerj.19426 (PMC12083466; doi:10.7717/peerj.19426)
Supplement: Supplemental Information 2 [file peerj-13-19426-s002.pdf]

# Questionnaire on
# Husbands' knowledge and attitudes regarding postpartum depression (coding and scoring)

**Part I: Husbands' Demographic Information:**

1. **Nationality**

    **1)** Saudi

    **2)** Non-Saudi

2. **Age**

    1) 20 -29

    2) 30 -39

    3) 40 -49

    4) 50 and more

3. **Region**

    1) Western region

    2) Southern region

    3) Central region

    4) Eastern region

    5) Northern region

4. **Education level**

    1) Primary school

    2) Intermediate school

    3) High school

    4) Bachelor degree / Diploma

    5) Higher than Bachelor degree

5. **Occupation**

    1) Government employee
    2) Private /business
    3) Free business
    4) Not working

6. **Monthly family Income**

    1) Less than 5000 riyals
    2) 5000-10000 riyals
    3) More than 10000 riyals

7. **Number of years of marriage?**

    1) 1 - 3 years
    2) 4 - 6 years
    3) More than 6 years

8. **Number of living children:**

    1) 1 child
    2) 2 children
    3) 3 children
    4) 4 children
    5) > 5 children

9. **Age of the youngest child?**

    1) 6 months and less
    2) 7-12 months
    3) >1-2 years
    4) More than 2 years

**10. Gender of the children?**

1) Only Males
2) Only Females
3) Both males and females

**11. Are you satisfied with your children gender?**

1) Yes
2) No

**Part II: Knowledge about PPD**

**Section 1: Previous information about PPD and experienced with PPD**

**1. You have previous information about postpartum depression?**

1) Yes
2) No

**2. If yes: What** the source of your information?

1) Website
2) Physicians/ nurses
3) Social media
4) Family / friends
5) TV /radio interviews
6) Others

**3. Had come across women with postpartum depression?**

1) Yes
2) No

**4. Has your wife ever been diagnosed with postpartum depression before?**

1) Yes
2) No

**Part II: Knowledge about PPD**

**Section 2: Knowledge about Causes and symptoms of PPD.**

✸ **Rating scale:** Yes (1) point and No / I don't know (0).

| **A. Causes of Postpartum Depression** | **YES (1)** | **NO (0)** | **I Don't know (0)** |
|---|---|---|---|
| **1. Genetic/hereditary** | ○ | ○ | ○ |
| **2. Crisis situation (Death of loved one, loss of job, divorce) in life** | ○ | ○ | ○ |
| **3. Poor relationship/ marital conflicts** | ○ | ○ | ○ |
| **4. History of depression** | ○ | ○ | ○ |
| **5. Lack of family support** | ○ | ○ | ○ |
| **6. Ghost possessed or doing sin or Blackmagic*** | ○ | ○ | ○ |
| **7. lack of confidence in taking care of baby** | ○ | ○ | ○ |
| **8. Health problem/sickness of baby** | ○ | ○ | ○ |
| **9. Domestic violence/husband violence** | ○ | ○ | ○ |
| **10. Older age (more than 20 years) *** | ○ | ○ | ○ |
| **11. Disappointment with gender of the baby** | ○ | ○ | ○ |
| **12. Poverty/financial difficulties** | ○ | ○ | ○ |
| **13. Poor education of the mother** | ○ | ○ | ○ |
| **14. Increased work pressure/stress** | ○ | ○ | ○ |
| **15. Personal strength *** | ○ | ○ | ○ |
| **16. Substance abuse (Alcohol) among husband** | ○ | ○ | ○ |
| **17. Single mother** | ○ | ○ | ○ |
| **B. Symptoms of Postpartum Depression** | **YES (1)** | **NO (0)** | **I Don't know (0)** |
| **1. Feeling sad/miserable** | ○ | ○ | ○ |
| **2. Mother bonding with baby *** | ○ | ○ | ○ |
| **3. Worry about bonding with baby** | ○ | ○ | ○ |

| | | | |
|---|---|---|---|
| **4. Interested in doing household chores *** | ○ | ○ | ○ |
| **5. Interested in talking with others *** | ○ | ○ | ○ |
| **6. Feeling of fatigue/weakness** | ○ | ○ | ○ |
| **7. Feeling stressed/anxious** | ○ | ○ | ○ |
| **8. Loss of interest/pleasure** | ○ | ○ | ○ |
| **9. Sleeping problems** | ○ | ○ | ○ |
| **10. Lack of confidence** | ○ | ○ | ○ |
| **11. Anger** | ○ | ○ | ○ |
| **12. Weight/appetite changes** | ○ | ○ | ○ |
| **13. Irritability** | ○ | ○ | ○ |
| **14. Crying spells for no reason** | ○ | ○ | ○ |
| **15. Death wishes** | ○ | ○ | ○ |
| **16. Postpartum depression may occur after birth to 6 months only*** | ○ | ○ | ○ |
| **Key:** <br> *** Revise coded** **(Negatively worded)** | | | |

**Part III: Husbands attitudes toward Postpartum Depression**

✸ **Rating scale: Agree (3) point, partly agree (2) and Disagree (1).**

| **Attitudes towards Postpartum Depression** | **Agree (3)** | **partly agree (2)** | **Disagree (1)** |
|---|---|---|---|
| **1.** I feel shame and do not tell anyone that my relative has postpartum depression. * | ○ | ○ | ○ |
| **2.** Postpartum women who have postpartum depression cannot be good mothers. * | ○ | ○ | ○ |
| **3.** Postpartum women who have postpartum depression should stay at home. * | ○ | ○ | ○ |
| **4.** We should be patient and have empathy with the women who have postpartum depression. | ○ | ○ | ○ |
| **5.** Postpartum women who have postpartum depression cannot take care of her own children. * | ○ | ○ | ○ |
| **6.** Postpartum depression is an uncured disease and will continue increasing in severity. * | ○ | ○ | ○ |
| **7.** Postpartum women who have postpartum depression are a pitiable person. | ○ | ○ | ○ |
| **8.** I feel it oppressive to take care of a woman who has postpartum depression. * | ○ | ○ | ○ |
| **9.** Postpartum women who have postpartum depression cannot make decisions at all. * | ○ | ○ | ○ |
| **10.** Postpartum women who have postpartum depression should not have another child. * | ○ | ○ | ○ |
| **11.** I am ready to help if my relative has postpartum depression. | ○ | ○ | ○ |
| **12.** Postpartum women who have postpartum depression is a burden to the family. * | ○ | ○ | ○ |
| **13.** Postpartum women who have postpartum depression should be treated in hospital. | ○ | ○ | ○ |
| **14.** Postpartum depression is an alert sign that a postpartum woman needs help from a caregiver. | ○ | ○ | ○ |

| | | | |
|---|---|---|---|
| **15.** If a woman develops postpartum depression, mother and baby to be separated. * | ○ | ○ | ○ |
| **16.** Postpartum depression does not require any treatment. * | ○ | ○ | ○ |
| **17.** Postpartum depression is common among women. | ○ | ○ | ○ |
| **18.** Breast feeding to be stopped if a woman develops postpartum depression. * | ○ | ○ | ○ |
| **19.** Woman with postpartum depression have a high risk of committing suicide. * | ○ | ○ | ○ |

**Key:**

*** Revise coded (Negatively worded)**
